# Implantation of customized 3-D printed titanium prosthesis in limb salvage surgery: a case series and review of the literature

**DOI:** 10.1186/s12957-015-0723-2

**Published:** 2015-11-04

**Authors:** Hongbin Fan, Jun Fu, Xiangdong Li, Yanjun Pei, Xiaokang Li, Guoxian Pei, Zheng Guo

**Affiliations:** Department of Orthopaedic Surgery, Xi-Jing Hospital, Fourth Military Medical University, West Chang-le Road, Xi’an, 710032 China

**Keywords:** Rapid prototyping, Prosthesis, Titanium, Electron beam melting

## Abstract

**Background:**

Although modular prosthesis is commercially available to meet requirements in most limb salvage surgeries, customized prosthesis is still needed. In contrast to traditional complicated procedures, rapid prototyping (RP) technique can directly manufacture customized titanium prosthesis. The objectives of this study were to describe the workflow of this technique and show the follow-up results of patients.

**Methods:**

Three patients with clavicle Ewing’s sarcoma (ES), scapular ES, and pelvic chondrosarcoma (CS) were scanned by computer tomography (CT). The images were segmented and reconstructed for preoperative planning and prosthesis design. Then, the data of prosthesis were imported into an electron beam melting system to manufacture implants. These three patients received prosthesis implantation after tumor excision. They were followed up to evaluate survival rate, functional outcome, and complications.

**Results:**

All patients were alive with no evidence of disease. The Musculoskeletal Tumor Society (MSTS) scores were 93, 73, and 90 % for patients with clavicle ES, scapular ES, and pelvic CS, respectively. No surgical complications including limb length discrepancy, screw loosening, and implant breakage were observed in current study.

**Conclusions:**

Electron beam melting (EBM) is a useful method to directly manufacture customized titanium prostheses. It might improve the effectiveness of limb salvage surgery for sarcomas in unusual sites.

## Background

In the past several decades, various reconstructions including autograft, allograft, and customized or modular prosthesis have been extensively used for limb salvages [1]. Each has its own advantages and limitations. Autograft has superior healing capability due to excellent osteoconductive and osteoinductive properties. However, it requires invasive collection and supply is limited [2]. Allograft can supply an immediate structural support as well as an anchor for reattachment of ligament and muscle. The associated disadvantages include possible disease transmission and slow incorporation into the host bone [3]. In addition, both autograft and allograft have to be manually carved to precisely fit the defects. This process is usually time-consuming and laborious. Prosthesis can provide an immediate support and rapid return to weight bearing, but it is associated with frequent complications such as aseptic loosening, infection, and periprosthetic fracture [4]. Therefore, the key issue is to weigh up the pros and cons of each reconstruction for choosing the most suitable one.

The original prosthesis is custom-made, which might cause some delay in treatment. In recent years, the modular prosthesis has been developed and extensively used in limb salvage surgeries due to being readily available. The studies indicated that the mean Musculoskeletal Tumor Society (MSTS) 93 score ranged from 57.2 to 62.0 % after modular hemi-pelvic prosthesis reconstruction [5, 6]. If customized prostheses were implanted, the patients showed similar functional scores (range 50 to 59.4 %) [7, 8]. For proximal femoral reconstruction, the estimated 5-year modular prosthesis survival rate was 90.7 % and the mean Toronto Extremity Salvage (TES) score was 61 % [9]. On the other hand, the survival of customized prosthesis was 77 % at 10 years and 57 % at 20 years [10]. Therefore, modular prosthesis can provide versatile reconstructions and acceptable functions for patients with sarcomas of extremity and pelvis.

However, there is no available modular prosthesis to reconstruct the clavicle, scapula, and ilium. After excisions of these bones, the optimal reconstruction has not yet been determined even though various techniques have been developed. Massive bone grafting (allograft or recycled autograft) could be an ideal biological reconstruction. Nevertheless, there remain distinct concerns including bone absorption, fracture, infection, and immune rejection [11, 12]. Prosthesis is another promising approach to restore postoperative function [13, 14]. Besides the requirement of demanding surgical techniques, the key problem of this approach is the limited availability of suitable prostheses. The most modern prosthesis is produced by machining solid titanium block, which was followed by applying different surface and geometry treatments to improve stability and osteointegration. However, none of these conventional techniques is capable of producing a completely controlled implant geometry and surface morphology in only one step.

The rapid prototyping (RP) is a process which directly generates physical objects with defined structure and shape on the basis of virtual mode data. The commonly used RP techniques include stereolithography (SLA), selective laser sintering (SLS), fused deposition modeling (FDM), laminated object manufacturing (LOM), inkjet printing, and electron beam melting (EBM). EBM is a kind of 3-D printing technique. It can be used to fabricate metallic components with complex shapes and porous structures [15]. The irregular bones (clavicle, scapula, and ilium) have unique dimensions. Therefore, it may be a feasible solution to design customized prosthesis and manufacture it rapidly. Ti-6Al-4V (TAV) alloy scaffolds manufactured by EBM have shown complete osteointegration within 60 days in animal study [16]. In patients with maxillofacial reconstruction, TAV implants manufactured by EBM were used to repair the zygomatico-orbital defects. The individual digital planning procedures, rapid prototyping, and titanium implants were proved to be effective [17]. However, few studies focus specifically on the application of customized 3-D-printed TAV prosthesis in limb salvage surgeries until now. There is also a lack of information on the prosthesis survival, functional outcomes, and complications.

In this study, we designed the customized titanium prosthesis through a 3-D virtual model based on the medical image data. Then, the prosthesis was manufactured by EBM and implanted to fill the osseous defect after tumor resection. The objectives of this study were (1) to describe the workflow of this technique and (2) to show the follow-up results of patients with prosthesis. A review of the literature on this subject was also provided.

## Methods

### Image acquisition and prosthesis design

Firstly, all patients were scanned by computer tomography (CT) (Mutislice 64, GE Healthcare, USA). Magnified slices with 0.625 mm thickness were obtained using a soft tissue standard filter (a matrix of 512 × 512 pixels) and stored in Digital Imaging and Communication in Medicine (DICOM) format. The process of image segmentation was conducted using the Mimics software (Leuven, Belgium). The bone was isolated from other tissues and structures such as muscle, fat, skin, and metal table of CT scanner. Pseudo color image was applied on DICOM image pixel data, and segmented images were used to reconstruct a 3-D model. Because the tumor invasion usually destroyed the partial or total structures of affected bones, the 3-D tumor models appeared to be incomplete. It was not suitable for prosthesis design. In this scenario, the images of contralateral anatomical site were used to create a 3-D mirror model for prosthesis design. Under virtual condition, the tumor was simulated to be excised and the prosthesis was fixed at the defect site. The authors could rotate the 3-D model to observe and refine the prosthesis design until the most appropriate one was selected. In comparison to stainless steel (190 GPa) and cobalt-chrome (230 GPa), titanium (110 GPa) is considered a low modulus metal. However, its modulus is still six times greater than cortical bone (7–30 GPa). This mismatch in Young’s modulus creates a stress-shielding effect [18]. To solve this problem, the porous structure was introduced into the titanium prosthesis design to reduce the modulus. Finally, the safe margin, cutting plane, implant position, and screw sites were preoperatively planned and marked on 3-D model basing on surgical approach and exposure.

### Manufacturing the TAV prosthesis

**Fig. 1:**
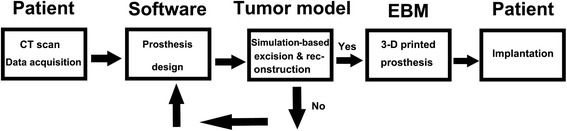
The workflow of 3-D printed prosthesis implantation

The porous TAV prosthesis was manufactured by EBM technique according to the following procedures. After the prosthesis design was completed, the data were saved in standard template library format and imported into EBM S12 system (Arcam AB, Sweden). Then, the TAV alloy powder was melted layer by layer in the EBM system. The structure of prosthesis was remolded according to the computer-aided design (CAD) model. Thereafter, the residual powder in prosthesis was removed by acid treatment and ultrasonic cleaning. Finally, the prosthesis was sterilized and packed for further use. The workflow of 3-D printed prosthesis implantation is shown in Fig. 1.

### Patient information

From November 2011 to June 2012, three female patients with malignant tumors received operative treatments in our department. X-ray, CT, magnetic resonance imaging (MRI), and single-photon emission computed tomography/CT (SPECT/CT) scan were performed on all patients to determine the tumor margin, tumor stage, and cutting plane. All patients underwent needle biopsy before surgery to obtain pathological diagnoses. The preoperative diagnoses were clavicle Ewing’s sarcoma (ES) in one patient, scapular ES in one, and pelvic chondrosarcoma (CS) in one. Adjuvant chemotherapy was administrated in patients with ES. The study was approved by the institutional review board of Xi-Jing Hospital, Fourth Military Medical University (FMMU). All patients provided their written informed consent, which was recorded and saved by the staff of the ethics committee of Xi-Jing hospital.

### Follow-up and survival

Local recurrence, metastasis, functional results, and complications were followed up every 3 months for the first two years, then 6 months for the third year. The plain radiographs, chest CT scan, MRI, and bone scintigraphy scan were used for follow-up purposes. The functions were evaluated by MSTS93 functional score [19].

### Case series

#### Patient 1

**Fig. 2:**
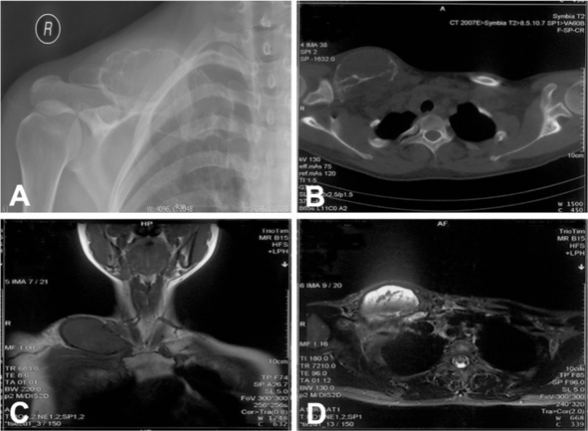
A 21-year-old woman with clavicle Ewing’s sarcoma. **a** X-ray film showed osteolytic lesion at the right clavicle. **b** CT showed large lytic lesion involving the whole clavicle. **c** The tumor showed low intensity on T1-weighted MRI. **d** The tumor showed hyper intensity on T2-weighted MRI

**Fig. 3:**
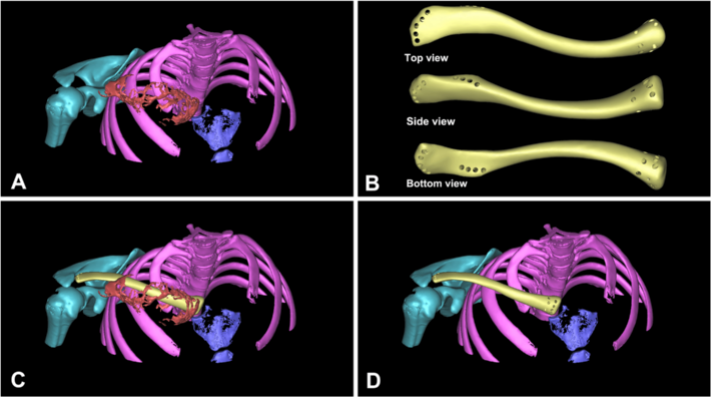
**a** Reconstructed 3-D clavicle tumor model. **b** The virtual 3-D model of prosthesis. **c** Simulated overlap of tumor and prosthesis. **d** The virtual clavicle reconstruction

**Fig. 4:**
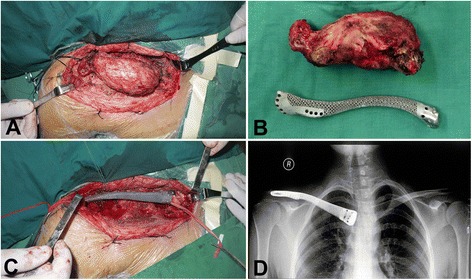
The clavicle tumor resection and prosthetic reconstruction. **a** Exposure of Ewing’s sarcoma. **b** The size of excised tumor and prosthesis was well matched. **c** Implantation and fixation of prosthesis. **d** X-ray showed proper placement and clavicular symmetry at 24 months postoperatively

A 21-year-old woman presented with a 6-month history of gradually increasing swelling of the clavicle. X-ray and CT scan showed a large expandable osteolytic lesion involving the whole clavicle (Fig. 2a, b). The tumor showed low intensity on T1-weighted MRI and hyperintensity on T2-weighted MRI (Fig. 2c, d). Staging study did not found evidence of metastasis. The biopsy revealed the diagnosis of clavicle ES. The patient received neoadjuvant chemotherapy as per the existing hospital protocol. Surgery was performed between week 8 and 10 after initiation of chemotherapy. Thereafter, chemotherapy was continued. Before operation, all image date were imported into Computer Assisted Operation System (CAOS) and surgical planning was made on a 3-D tumor model (Fig. 3a). The clavicle prosthesis was designed according to images of the contralateral site. The virtual 3-D models of prosthesis (Fig. 3b), overlap of tumor and prosthesis (Fig. 3c), and final reconstruction (Fig. 3d) were generated for surgical planning. The prosthesis was manufactured by EBM. During operation, the lesion was exposed and whole claviculectomy was performed with tumor-free margin (Fig. 4a). The size of prosthesis and excised clavicle was well matched. In addition, it had porous structure to reduce the modulus (Fig. 4b). It was implanted and fixed to the acromion, sternum, and coracoclavicular ligament with non-absorbable suture through the small holes on prosthesis (Fig. 4c). X-ray showed proper placement and clavicular symmetry at 24 months postoperatively (Fig. 4d).

#### Patient 2

**Fig. 5:**
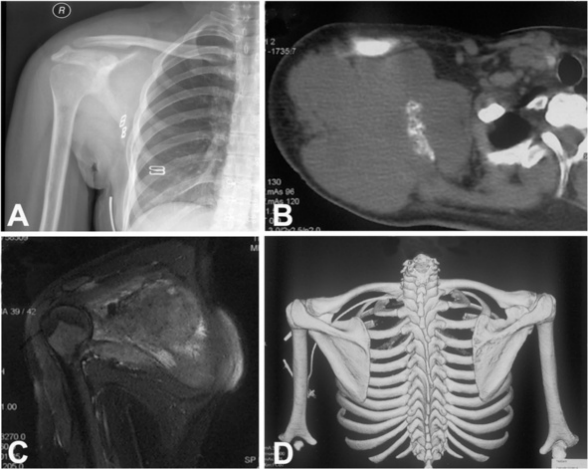
A 35-year-old woman with right scapular ES. **a** X-ray film showed osteolytic lesion with a bulky soft tissue shadow at right scapula. **b**, **c** CT and MRI showed destructed scapula and surrounding soft tissue mass. **d** 3-D CT reconstruction of affected scapula

**Fig. 6:**
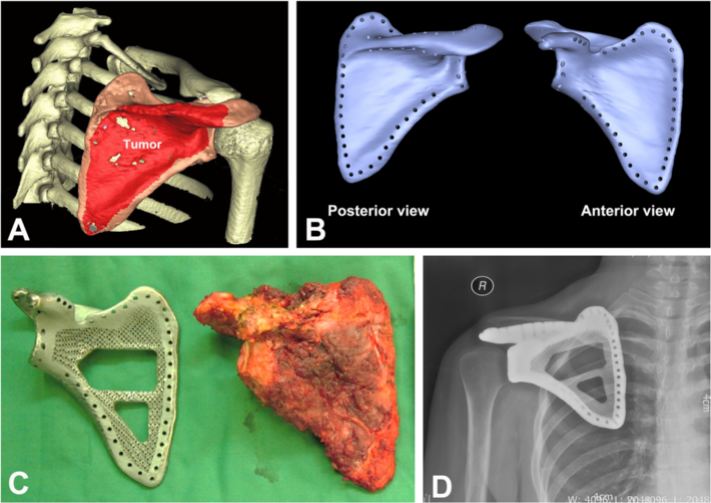
**a** Reconstructed 3-D scapular tumor model. **b** The virtual 3-D model of scapular prosthesis. **c** The size of excised tumor and prosthesis was well matched. **d** X-ray showed a proper shoulder articulation at 21 months postoperatively

A 35-year-old woman was diagnosed with right scapular ES after core needle biopsy. Staging studies, including plain film, MRI, chest CT scan, and full body scintigraphy, were performed. She was non-metastatic at presentation. The radiograph showed an osteolytic lesion with a bulky soft tissue shadow (Fig. 5a). Axial CT and coronal MRI images indicated that the tumor originated from the scapula had extended into surrounding muscles and formed a large soft tissue mass (Fig. 5b, c). The “moth-eaten” or mottled appearance of bone destruction was observed in 3-D CT reconstruction (Fig. 5d). The patient’s image data were imported into CAD system, and a 3-D tumor model was reconstructed (Fig. 6a). The size-matched prosthesis was designed according to data of the contralateral site (Fig. 6b). Before operation, she received new adjuvant chemotherapy. Then, the scapula was removed en bloc by an intra-articular incision of the glenohumeral joint (Malawer Type III resection) [20]. The size of excised scapula was similar to that of a porous TAV prosthesis manufactured by EBM (Fig. 6c). During the operation, major blood vessels and nerves to the upper extremity were preserved. The prosthesis was implanted and stabilized by wrapping a non-absorbable tape around the patient’s clavicle and prosthesis. The remnant of the coracoclavicular ligament was anchored back to the small holes on the coracoid process of the prosthesis with non-absorbable suture. The remaining surrounding muscles such as the trapezius, latissimus dorsi, deltoid, etc., were sutured back to small holes on the wings of the prosthesis. The humeral head was suspended by suturing the remaining rotator cuff and biceps tendon to the clavicle and neighboring muscles. X-ray showed a proper articulation at 21 months postoperatively (Fig. 6d).

#### Patient 3

**Fig. 7:**
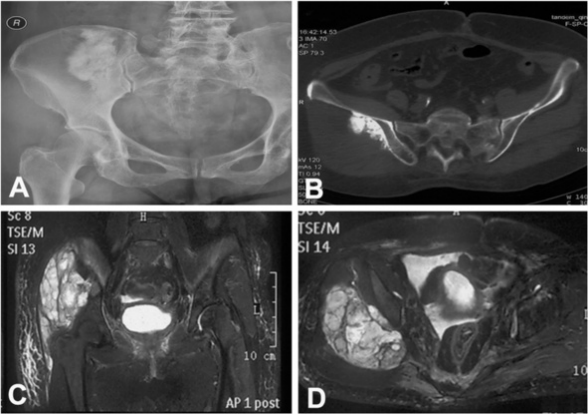
A 56-year-old woman with pelvic chondrosarcoma. **a** X-ray film showed a mixed lytic-sclerotic lesion of the right ilium. **b** CT showed a large soft tissue mass with scattered calcification. **c**, **d** Coronal and axial images of T2-weighted MRI showed the tumor involved in the right ilium and juxta-acetabular bone

**Fig. 8:**
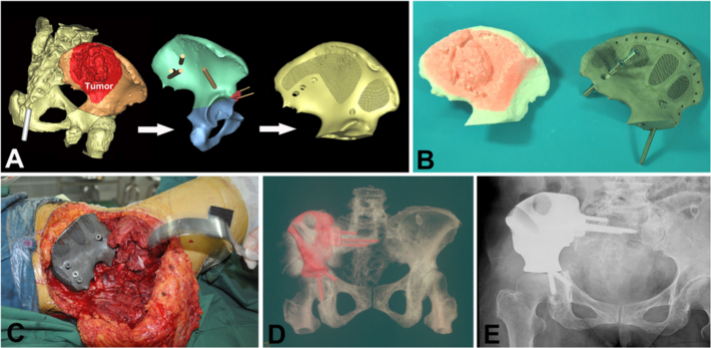
**a** Reconstructed 3-D pelvic tumor model and prosthesis design. **b** The porous prosthesis and simulated tumor excision. **c** Implantation and fixation of prosthesis. **d**, **e** 3-D CT reconstruction and X-ray film showed good alignment at 18 months postoperatively

A 56-year-old woman had a gradually enlarging pelvic mass for 5 months. The biopsy confirmed the diagnosis of pelvic CS. The plain radiograph showed a mixed lytic-sclerotic lesion on the right ilium (Fig. 7a). The CT scan revealed a large soft tissue mass with punctate or stippled calcification in it (Fig. 7b). The tumor had high signal intensity on T2-weighted MRI. It involved the right ilium and juxta-acetabular bone (Fig. 7c, d). All image data were imported into CAD and CAOS system for 3-D tumor model reconstruction and prosthesis design. Under virtual condition, the prosthesis was simulated to be implanted after tumor excision. The cutting plane, implant position, and screw sites were marked on 3-D model preoperatively (Fig. 8a, b). TAV alloy powder was melted in EBM system to manufacture prosthesis. The navigation-guided surgical resection was performed as previously reported [3]. After appropriate registration and calibration, the surgeons located and marked the anatomic position of intended bone cutting plane with K-wires by navigation tools as planned preoperatively. Then, the tumor was removed en bloc with an oscillating saw. After osteoectomy, the prosthesis was fixed to the rest pelvis and sacrum using screws (Fig. 8c). Plain radiograph and 3-D CT reconstruction showed stable fixation and good alignment at 18 months postoperatively (Fig. 8d, e).

## Results

### Case series

The average operation time was 92 min (65, 80, 130 min) and average blood loss was 500 ml (200, 500, 800 ml). There was no neurovascular bundle injury that occurred during surgery. The follow-up time was 24, 21, and 18 months in patients with clavicle ES, scapular ES, and pelvic CS, respectively. No local recurrence or metastasis was observed in these three patients. At their latest follow-up, all patients were alive with no evidence of disease (ANED). The range of motion (ROM) of the shoulder and hip joint is summarized in Table 1. According to MSTS 93 system for the upper and lower limb, the scores were 93, 73, and 90 % in patients with clavicle ES, scapular ES, and pelvic CS, respectively (Table 2). There were no limb length discrepancy, screw loosening, implant breakage, and joint collapse in any of the patients.

**Table 1:** Demographic and clinical data

Case no.	Gender/age (years)	Diagnosis (stage)	Margin	Follow-up (months)	Oncologic outcome			Joint ROM		
					Relapse	Metastasis	Patient status	Flex	Ext	Abd
1	F/21	Clavicle ES	Wide	24	None	None	ANED	90°	35°	80°
2	F/35	Scapular ES	Wide	21	None	None	ANED	55°	20°	35°
3	F/56	Pelvic CS (IIB)	Wide	18	None	None	ANED	120°	10°	30°

*ES* Ewing’s sarcoma, *CS* chondrosarcoma, *ANED* alive with no evidence of disease

**Table 2:** Functional outcome

Case no.	Gender/age (yrs)	Diagnosis	Pain	Function	Emotional acceptance	Supports/hand position	Walking/manual dexterity	Gait/lifting ability	MSTS score
1	F/21	Clavicle ES	5	4	5	5	5	4	28 (93 %)
2	F/35	Scapular ES	5	3	4	2	5	3	22 (73 %)
3	F/56	Pelvic CS	5	4	5	5	4	4	27 (90 %)

*ES* Ewing’s sarcoma, *CS* chondrosarcoma

### Literature review

PubMed search did not retrieve large studies on clinical application of customized prosthesis manufactured by RP technique. The potential advantage of RP technique lies within the possibility of manufacturing the complicated customized prostheses in greatly reduced time. Although the standard prosthesis is commercially available to meet the requirements in most surgical procedures, customized prosthesis is still needed in some cases. The indications for customized prosthesis are as follows: (1) patients outside the standard range with respect to implant size, implant morphology, or disease-specific special requirements; and (2) improved surgical outcome because of individual fitting and adequate match with individual anatomical needs [15].

The RP technique has been applied in reconstructing various anatomical structures especially in craniomaxillofacial surgery. In this scenario, a customized medical implant and surgical resection template is accurately designed using a CAD technique. The physical model of the individual implant, template, or skull replica can be produced through RP process. The CAD data of prosthesis are imported into RP machine to fabricate the physical object. The stereolithography apparatus prosthesis pattern is directly used in investment casting for production of TAV prosthesis. Singare et al. [21] reported two patients with large maxillary bone defects received customized prostheses implantation. The prosthesis perfectly fitted the defects during the operation and surgery time was greatly reduced. D’Urso et al. [22] reviewed the outcome of 30 patients who underwent cranioplasty surgeries. Data acquired from CT were used to manufacture exact plastic replicas of craniotomy defects and cranioplastic implants using RP technology. The 3-D mirror imaging was used to extrapolate existing anatomy to design implants. The customized prosthesis could reduce operating time and afford excellent cosmesis. Cho et al. [23] described the application of 3-D printing technology to fabricate a mirror-image single crown of a maxillary central incisor. A 73-year-old patient with a recently replaced metal ceramic crown had discomfort due to the non-anatomic lingual contour of the crown. With CAD software and RP technology, the shape of the contralateral central incisor was duplicated and reproduced to make a mirror-image for a new crown.

Customized prosthesis manufactured by RP technique was also applied in limb salvage surgeries. Wang et al. and Li et al. [24, 25] designed a customized hemi-knee joint trying to solve the problem of cartilage necrosis in hemi-joint allograft transplantation. Based on extracted 3-D contour images of articular cartilage, the artificial knee was designed with Surfacer 9.0 software. The 3-D contours of femoral condyle and subchondral bone were used to design the outer and inner surface of prosthesis, respectively. After the data were imported, the positive mold was fabricated by RP machine. It was used to manufacture prosthesis through mold-melted founding process. In combination with allograft, the prosthesis was used to treat the large defect after excision of distal femur sarcoma. It well matched the subchondral contour of the femoral condyle and provided an efficient way to prevent necrosis of allogenic cartilage in hemi-knee reconstruction. At 18 months postoperatively, the patient demonstrated satisfactory knee joint function. Dai et al. [26] reported their experience in customized hemi-pelvic prostheses implantation in ten patients who underwent internal hemi-pelvectomy for extensive pelvic tumors. The CT data were converted into stereolithographic file and processed by Magic RP software. The images of lesion and contralateral normal site were viewed and evaluated. Then, the data were exported to a RP machine and a pelvic model was made. After the simulated bone resection was done on the model, the hemi-pelvic prosthesis was designed and manufactured. The simulated installation and revision were performed on the model. The prosthesis was then implanted to reconstruct hip function. Except that four patients died of disease within 6 to 10 months postoperatively, the remaining six patients had good hip function after follow-up ranging from 21 to 48 months. However, the prostheses in these reports were still manufactured through mold-melted founding process. The RP technique was only used to fabricate positive mold. There was still no clinical report on manufacturing prosthesis directly through 3-D printing machine.

## Discussion

Although amputation is the most commonly used treatment in the last century for large tumors involving the clavicle, scapula, and pelvis, limb salvage has now become the consensus procedure for most cases. This is mostly attributed to improvements in preoperative imaging evaluation, more effective neoadjuvant chemotherapy, and advances in surgical technique. The development of customized titanium alloy prosthesis has augmented the limb salvage surgery in recent years.

Even though it is biologically compatible, titanium has difficulties in molding, casting, and milling. The shaping of titanium onto a model is limited by the artistic aptitude of technician and can be unreliable. CNC milling is also limited by difficulties encountered when trying to replicate complex anatomy or internal features due to collision between the milling tool and the object. With the development of RP technique, the accurate production of prosthesis with complicated morphology and internal features has become possible [22]. Metal RP processes like EBM and selective laser sintering can directly manufacture patient-specific medical TAV implants and devices. In the current study, three kinds of prosthesis manufactured by EBM were implanted to repair the defects in unusual sites. Compared to traditional manufacturing process, EBM greatly simplifies the processing steps and considerably reduced fabricating time. It shows a promising tendency in manufacturing customized prosthesis. The Food and Drug Administration (FDA) of the USA has recently approved the production of certain products fabricated using Arcam’s EBM process. Besides fabricating customized implants, several companies, including Adler Ortho (Italy), have used EBM to manufacture some special structures of standard prosthesis. For example, the lattice structure of acetabular cup is crucial for osteointegration in hip replacement. But the traditional manufacturing methods are complicated and time-consuming. Now, EBM has been used to fabricate such structures. This direct metal fabrication technique can eliminate several process steps associated with traditional methods [27]. Also in artificial hip system, the stem is usually much stiffer than host bone and, over time, this can cause stress shielding, bone resorption, and implant loosening. Additive processes allow the structure of the stem to be optimized to match the stiffness, or flexibility, of the host bone, reducing stress shielding [28].

Both surgeons and patients have benefited from RP technique. Provision of a preoperatively customized prosthesis and 3-D model can allow the surgeons to assess implant fit preoperatively, evaluate fixation sites, obtain a valuable overview of the procedure, and reduce operation time. The patients are able to see the 3-D model and implant preoperatively, which improves their understanding of the procedure.

In this case series, reconstructions were performed in the clavicle, scapula, and ilium. Although good shoulder function was reported to be achieved without reconstruction after total claviculectomy, the authors indicated that restoration was still needed basing on the requirements of function and cosmesis. The clavicle can provide protection for the major vessels and nerves at the base of the neck. Likewise, it serves a cosmetic function by providing a graceful curve. It also works as a strut to hold the glenohumeral joint in the parasagittal plane and increase the range of motion. The patient showed good shoulder function (MSTS score 93 %) and satisfactory ROM (Flexion 90°, extension 35°, abduction 80°), which correlated with the results of other studies [11, 29]. Regarding to the total scapulectomy, it required not only joint resection but also wide excision of shoulder girdle muscles. Residual function is usually minimal and unsatisfactory. Although various reconstructions after total scapulectomy have been applied, such as prosthetic replacement, recycled bone grafts, or soft tissue reconstruction, the optimal reconstruction has not yet been determined because the amount of remaining muscle and rotator cuff following scapulectomy varies with each surgery. Therefore, preoperative evaluation of medical images to confirm safe margins of excision is mandatory. In the current case, the glenoid was affected by tumor. The shoulder girdle was reconstructed with the remaining soft tissue and scapular prosthesis, which restored glenohumeral articulation. In contrary to the commonly used reconstruction with constrained total scapula prosthesis, only the scapula was replaced with customized implant considering for sparing of enough muscles. Soft tissue reconstructions including humeral suspension, reattaching the rotator cuff, and suturing the biceps tendons onto the clavicle were performed. The patient obtained benefit from this reconstruction, and functions were acceptable in comparison with the results of another study [30]. Although scapular allograft reconstruction was reported with satisfactory outcomes, resorption of allograft was observed and remained a distinct concern [12]. Therefore, TAV scapular prosthesis was preferred in current study.

Our study also had several limitations. Firstly, it was limited by the short-term follow-up and lack of control for comparison purposes between different reconstruction methods. The authors can only provide the preliminary results to introduce this technique in the current study. Secondly, the 3-D-printed prosthesis is not widely used due to the following reasons: (1) the complicated approval process, (2) the high price, and (3) the low incidence of sarcoma in unusual anatomy site, where no commercial modular prosthesis can be used. It was difficult to obtain a large number of patients in one institution. The small sample size did not allow sufficient power to explore the advantages of RP techniques. Thirdly, the porous structure was introduced into the design of prosthesis in order to reduce the modulus. But the final modulus of implant was not tested in this study. In spite of these shortcomings, the current study still added further information on customized prosthesis manufactured by EBM.

## Conclusions

EBM, a metal RP process, was used to directly manufacture customized TAV prostheses in this study. With the aid of CAOS, the accurate excision and precise reconstruction were achieved. These implants fitted the patient-specific anatomy well. The follow-up results proved the safety and effectiveness of prostheses. Although this single-center study is small in scale and the follow-up time is short, the results still demonstrate the advantages of this technology.
